# Editorial: Advancing pediatric type 1 diabetes management: metrics, technologies, and prevention of complications

**DOI:** 10.3389/fendo.2026.1910094

**Published:** 2026-06-23

**Authors:** Bruno Bombaci, Marta Bassi

**Affiliations:** 1 Department of Human Pathology in Adult and Developmental Age “Gaetano Barresi”, University of Messina, Messina, Italy; 2 Department of Neuroscience, Rehabilitation, Ophthalmology, Genetics, Maternal and Child Health (DINOGMI), University of Genoa, Genoa, Italy

**Keywords:** adolescence, automated insulin delivery, children, continuous glucose monitoring, exogenous insulin antibody syndrome, telemedicine, time in range

## 1 Introduction

The management of type 1 diabetes (T1D) in children and adolescents has recently undergone significant transformation due to advancements in continuous glucose monitoring (CGM) and automated insulin delivery (AID) devices. New glycemic metrics, first introduced by the International Consensus on Time in Range (1), and later supported by international clinical practice guidelines (2), have redefined the concept of optimal glucose control. However, adoption of technologies remains uneven across countries and socioeconomic groups. Furthermore, differences in treatment approaches are increasingly shaped by specific clinical phenotypes. The studies in this Research Topic offer a multidimensional view of current pediatric T1D care, from epidemiology to technology and complication prevention.

## 2 Summary of articles

### 2.1 Mapping the burden

Using Global Burden of Disease data, Jin et al. analyze pediatric T1D in China from 1990 to 2021 and project trends through 2040. Their analysis highlights the growing burden in younger children and underscores the need for earlier recognition and stronger surveillance. Confirming previous global data (3), incidence is increasing most rapidly among the youngest children. Infants under one year also show rising incidence and a recent rebound in mortality, which is concerning when compared with patterns observed in high-income settings. Additionally, a sex disparity emerges, with girls showing a greater burden, including higher rates of incidence, mortality, and underdiagnosis. These findings reinforce the need for prompt recognition of signs and symptoms within public health systems, especially in rural regions where timely diagnosis may be more difficult.

### 2.2 Clinical presentation of T1D and acute complications

Two studies focused on clinical presentation and early inpatient course, highlighting how baseline phenotype may influence acute outcomes. Overweight and obesity affect approximately a third of children with T1D in the international SWEET registry (4), yet their impact on disease presentation remains underexplored. In a cohort of 994 Hungarian children, Muzslay et al. found that those with overweight presented with the most severe metabolic derangement at onset, and that overweight and obese children together had a higher prevalence of diabetic ketoacidosis (DKA) than their normal weight peers. This suggests that excess weight may contribute to a more severe metabolic presentation at diagnosis. Obese children, however, showed higher C-peptide levels, suggesting greater residual β-cell function. The higher risk of DKA despite preserved β-cell function might suggest that obesity-related inflammation and insulin resistance may contribute to acute metabolic decompensation.

Hypoglycemia remains one of the main concerns of insulin therapy in children, particularly during inpatient care (5). Li et al. then examined this Research Topic in 567 newly diagnosed children and adolescents, and found that 81.7% experienced at least one episode of hypoglycemia while hospitalized. Mild episodes clustered overnight, whereas more serious events were found predominantly before lunch, suggesting different mechanisms by severity. Younger age, longer hospitalization, combined regimens of insulin, and lower fasting C-peptide independently predicted hypoglycemia. These predictors may help identify children at higher risk and guide more targeted preventive strategies during hospitalization.

### 2.3 Technology: long-term data and equity

Four contributions examine technology and AID from complementary perspectives, spanning efficacy, equity, longitudinal outcomes, and education.

Jendle et al. report results from a multicenter randomized controlled trial comparing the use of the MiniMed 670G/770G system versus multiple daily injection regimens in adults and children. AID was associated with significant reductions in HbA1c in participants with elevated baseline values and time below range in those near target, low rates of acute complications, reinforcing its central role in contemporary T1D management and supporting its position as standard of care (6).

Figg et al. assess disparities with BEAD-T1D, a prospective mixed-methods study protocol investigating barriers and promoters to CGM and AID adoption among youth with public insurance in the US. Using a social-ecological model, it examines drivers at different levels influencing technology uptake. This study is particularly relevant because it addresses not only whether technology works, but also why access and uptake remain unequal.

Tarasiewicz et al. provide three years of longitudinal data on AID use following 50 children on the MiniMed 780G. Time in range peaked at six months after AID initiation and then declined modestly over time, along with a parallel increase of auto-correction boluses, suggesting that early benefits may partially attenuate without sustained follow-up and support. Nevertheless, both time in range and tight range (70–140 mg/dl), an emerging metric of clinical and research interest (7), remained within recommended targets at 36 months.

Nagla et al. also provided an important reminder that technology alone cannot be the solution. A structured self-management diabetes education program was given to 100 youth with diabetes using high-definition video in Egypt. Results included significant improvements in diabetes knowledge and reduction in HbA1c of 0.81%, with sustained benefits at 4 months. An independent association was found between improved knowledge and reduced HbA1c values. These results support the view that structured education is a therapeutic intervention in its own right, not merely an adjunct to technology, as does telemedicine, which can extend specialist support and help preserve glucose control when in-person care is disrupted (8).

### 2.4 Beyond the standard phenotype

A further contribution extended the discussion beyond standard T1D phenotypes. Zhang et al. described 5 cases of T1D complicated by exogenous insulin antibody syndrome, a rare complication characterized by high-titer antibodies against exogenous insulin and marked glucose variability. Their case series confirmed that CGM provides a more reliable assessment of glycemic instability than HbA1c alone, and suggested mycophenolate mofetil may be a useful option in refractory cases.

## 3 Conclusions

Taken together, these studies offer a representative picture of contemporary pediatric T1D research, from epidemiology to acute complications, technology, education, and rare phenotypes. They affirm the value of technology while also emphasizing that its benefits must reach all children, they show how weight status shapes the disease presentation and the risk of complications, and they restore education to its place as a core therapeutic tool. Progress in pediatric T1D does not come from a single innovation but from the coordinated advance of metrics, technologies, equity, education and prevention: the dimensions this Research Topic was conceived to address.

## References

[1] Battelino T Danne T Bergenstal RM Amiel SA Beck R Biester T . Clinical targets for continuous glucose monitoring data interpretation: Recommendations from the International Consensus on Time in Range. Diabetes Care. (2019) 42:1593–603. doi: 10.2337/dci19-0028 31177185 PMC6973648

[2] de Bock M Agwu JC Deabreu M Dovc K Maahs DM Marcovecchio ML . International society for pediatric and adolescent diabetes clinical practice consensus guidelines 2024: glycemic targets. Horm Res Paediatr. (2024) 97:546–54. doi: 10.1159/000543266 39701064 PMC11854972

[3] Norris JM Johnson RK Stene LC . Type 1 diabetes-early life origins and changing epidemiology. Lancet Diabetes Endocrinol. (2020) 8:226–38. doi: 10.1016/s2213-8587(19)30412-7 31999944 PMC7332108

[4] Maffeis C Birkebaek NH Konstantinova M Schwandt A Vazeou A Casteels K . Prevalence of underweight, overweight, and obesity in children and adolescents with type 1 diabetes: Data from the international SWEET registry. Pediatr Diabetes. (2018) 19:1211–20. doi: 10.1111/pedi.12730 30033651

[5] Ruan Y Moysova Z Tan GD Lumb A Davies J Rea RD . Inpatient hypoglycaemia: Understanding who is at risk. Diabetologia. (2020) 63:1299–304. doi: 10.1007/s00125-020-05139-y 32300821 PMC7286944

[6] Sherr JL Heinemann L Fleming GA Bergenstal RM Bruttomesso D Hanaire H . Automated insulin delivery: Benefits, challenges, and recommendations. A consensus report of the Joint Diabetes Technology Working Group of the European Association for the Study of Diabetes and the American Diabetes Association. Diabetologia. (2023) 66:3–22. doi: 10.1007/s00125-022-05744-z 36198829 PMC9534591

[7] Dunn TC Ajjan RA Bergenstal RM Xu Y . Is it time to move beyond TIR to TITR? Real-world data from over 20,000 users of continuous glucose monitoring in patients with type 1 and type 2 diabetes. Diabetes Technol Ther. (2024) 26:203–10. doi: 10.1089/dia.2023.0565 38444315 PMC10877396

[8] Lombardo F Salzano G Bombaci B Basile P Lucania G Alibrandi A . Has COVID-19 lockdown improved glycaemic control in pediatric patients with type 1 diabetes? An analysis of continuous glucose monitoring metrics. Diabetes Res Clin Pract. (2021) 178:108988. doi: 10.1016/j.diabres.2021.108988 34331977 PMC8416096

